# Reduced Slow‐Wave Sleep and Sleep‐Disordered Breathing Are Linked to Suicidal Ideation in Major Depressive Disorder

**DOI:** 10.1002/brb3.71693

**Published:** 2026-08-17

**Authors:** Limin Yang, Yu Wang, Renyun Zhang, Shilong Jia, Xuefei Wu, Yun'ai Su, Jitao Li

**Affiliations:** ^1^ Shandong Mental Health Center Shandong University Jinan China; ^2^ Peking University Sixth Hospital, Peking University Institute of Mental Health, NHC Key Laboratory of Mental Health (Peking University), National Clinical Research Center for Mental Disorders (Peking University Sixth Hospital) Beijing China

**Keywords:** major depressive disorder, polysomnography, sleep‐disordered breathing, slow‐wave sleep, suicidal ideation

## Abstract

**Background:**

Suicidal ideation (SI) is common in major depressive disorder (MDD) and has been linked to sleep disturbances. However, the relative contributions of subjective sleep quality and objective sleep architecture to SI remain unclear.

**Methods:**

A total of 103 inpatients with MDD underwent polysomnography (PSG) and completed the Pittsburgh Sleep Quality Index (PSQI). Based on HAMD‐17 item 3 scores, participants were classified into SI (*n* = 49) and non‐SI (N‐SI, *n* = 54) groups. Clinical characteristics, subjective sleep quality, and PSG‐derived sleep parameters were compared between groups. Binary logistic regression was performed to identify independent predictors of SI.

**Results:**

Compared with the N‐SI group, patients with SI had longer illness duration, more depressive episodes, higher depressive severity, and poorer subjective sleep quality. PSG analyses revealed that SI patients showed significantly lower N3% and higher apnea–hypopnea index (AHI). Logistic regression indicated that PSQI score, N3%, and AHI were independent predictors of SI. A combined model incorporating these three factors demonstrated excellent discriminative ability (AUC = 0.924) for identifying SI.

**Conclusions:**

Both subjective sleep disturbance and objective sleep abnormalities, specifically reduced slow‐wave sleep and elevated AHI, are strongly associated with SI in MDD. Integrating subjective and objective sleep assessments may enhance suicide risk detection and guide targeted interventions for prevention.

## Graphical abstract

Graphical abstract illustrating that sleep abnormalities associated with suicidal ideation in major depressive disorder.

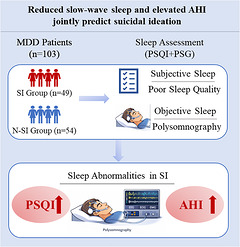

**Abbreviations**
AHI: apnea–hypopnea index
MDD: major depressive disorder
N‐SI: no suicidal ideation
PSQI: Pittsburgh Sleep Quality Index
SI: suicidal ideation

## 1 Introduction

Each year, nearly 900,000 people worldwide die by suicide, making it a critical challenge in global public health (World Health Organization 2014). Major depressive disorder (MDD), a leading contributor to the global disease burden (COVID‐19 Mental Disorders Collaborators 2021), is strongly linked to suicidality. Individuals with MDD face an approximately 20‐fold higher risk of suicide than the general population (Chesney et al. 2014), highlighting the urgent need to identify modifiable risk factors.

Suicidal ideation (SI) is among the most common and clinically significant symptoms of MDD, with prevalence rates from 18% to 58% in patients (Sokero et al. 2003). SI serves as a major predictor of suicide attempts and deaths and is closely associated with more severe depressive symptoms, greater psychiatric comorbidity, poorer neuropsychological functioning, and reduced quality of life (Borentain et al. 2020; Roy et al. 2017; Su et al. 2023). Importantly, SI has been linked to poorer antidepressant treatment response and lower remission rates (Lopez‐Castroman et al. 2016), underscoring its role as a key prognostic marker for adverse treatment outcomes and elevated suicide risk.

Sleep disturbance has been consistently identified as a correlate of SI in MDD (Bernert et al. 2014; Gunnell et al. 2013). In particular, insomnia symptoms reliably predict SI across both cross‐sectional and longitudinal studies (Harris et al. 2020; Liu et al. 2020). A meta‐analysis showed that insomnia doubles the risk of SI (Harris et al. 2020) and serves as a robust predictor of suicidal thoughts and behaviors (Bernert et al. 2015; Pigeon et al. 2012). Moreover, insomnia predicts SI trajectories and remains a significant predictor even after accounting for other psychological factors (Ribeiro et al. 2012). Collectively, these findings emphasize that sleep disturbance represents a critical and potentially modifiable factor influencing suicide risk in MDD.

Although subjective sleep disturbances are linked to SI, objective studies using polysomnography (PSG) remain limited and inconsistent. Prior PSG research suggests that rapid eye movement (REM) sleep abnormalities, such as increased REM percentage, heightened REM activity, or prolonged REM duration, are associated with SI and suicidal behavior (Agargun and Cartwright 2003), while reduced sleep efficiency has been linked to more lifetime suicide attempts (Hiltunen et al. 2011; Sabo et al. 1991; Verkes et al. 1996). However, few studies have systematically examined stage‐specific sleep parameters in relation to current SI.

To address these gaps, this study investigates the association between objective sleep architecture, including non‐rapid eye movement sleep (NREM) and REM sleep, and current SI in patients with MDD. By precisely quantifying sleep stages, we aim to clarify how specific abnormalities in sleep architecture contribute to SI. The findings may inform sleep‐focused interventions and help identify high‐risk patients, ultimately supporting efforts to reduce suicide risk.

## 2 Material and Methods

### 2.1 Participants

A total of 103 inpatients with major depressive disorder were recruited from the Shandong Mental Health Center between January 2023 and December 2024. The study was approved by the Ethics Committee of the Shandong Mental Health Center (Approval No.: KYSJWLL2024‐1‐087) and conducted in accordance with the Declaration of Helsinki (World Medical Association 2013). All participants provided written informed consent.

Inclusion criteria were as follows: (1) aged 18–60 years; (2) meeting the diagnostic criteria for major depressive disorder as defined by the Diagnostic and Statistical Manual of Mental Disorders, Fifth Edition (DSM‐5); (3) a 17‐item Hamilton Depression Rating Scale (HAMD‐17) score ≥ 20; (4) a Pittsburgh Sleep Quality Index (PSQI) score ≥8, the validated cutoff score for poor sleep quality in Chinese populations (Guo 2013); and (5) no use of antidepressants, benzodiazepines, or other sedative–hypnotic agents within the preceding month. Exclusion criteria included severe physical or organic brain disease, a history of psychoactive substance abuse, pregnancy or breastfeeding, or receipt of modified electroconvulsive therapy within the past three months.

### 2.2 Clinical and Cognitive Assessments

Depressive symptoms were assessed using the HAMD‐17, with item 3 (D3) evaluating suicidal ideation (D3> 1 indicating presence; D3 = 0 indicating absence). Sleep quality over the past month was assessed using the Pittsburgh Sleep Quality Index (PSQI), comprising 19 self‐rated and 5 clinician‐rated items across seven components, each scored 0–3 (total 0–21, higher scores indicating poorer sleep).

### 2.3 Inpatient Polysomnography

Overnight polysomnography (PSG) was performed on the night of admission using the Philips Respironics multi‐channel system. Participants abstained from alcohol, caffeine, and sleep‐affecting medications for 24 h prior, and sleep was recorded for ≥7 h. Trained personnel applied and removed the equipment, and the following parameters were collected: time in bed (TIB), total sleep time (TST), sleep latency (SL), sleep efficiency (SE), REM latency, NREM (N1‐N3) and REM sleep percentages (%), total number of awakenings (NWAK), arousal Index (AI), and apnea–hypopnea index (AHI). Recordings were manually scored by trained technicians according to American Academy of Sleep Medicine (AASM) criteria (Ballard et al. 2016).

### 2.4 Statistical Analysis

Statistical analyses were performed using SPSS 26.0. Continuous variables were presented as mean ± standard deviation (SD) and compared between groups using independent‐samples *t*‐tests, or the Mann–Whitney *U* test for non‐normally distributed data. Categorical variables were expressed as *n* (%) and analyzed using χ^2^ tests. Skewed PSG‐derived sleep parameters were log‐transformed (including SL, REM Latency, SE, NWAK, AI, and AHI), and age, sex, duration of the current episode, number of episodes, past SI history, and family history were included as covariates in group comparisons. To identify sleep‐related predictors of suicidal ideation (SI; SI present = 0, SI absent = 1), logistic regression analyses were conducted including differing PSG parameters. Bootstrap resampling (1000 iterations) was used to obtain robust standard errors and 95% bias‐corrected confidence intervals. ROC curves were generated in R using the pROC and ggplot2 packages. All tests were two‐tailed, with *p* < 0.05 considered statistically significant.

## 3 Results

### 3.1 Demographic and Clinical Characteristics of Participants

Among 109 initially enrolled patients with MDD, 6 did not complete PSG, resulting in a final analytic sample of 103 patients. Based on HAMD‐17 item 3 (D3 ≥ 1), 49 patients (47.6%) were classified into the SI group, and 54 patients (52.4%) into the N‐SI group. The demographic and clinical characteristics of the participants are presented in Table 1. There were no significant group differences in age, sex, or educational level (all *p* > 0.05). Compared with N‐SI group, patients in the SI group showed a longer duration of the current episode (*p* = 0.001), a higher number of depressive episodes (*p* = 0.048), a greater prevalence of prior SI (*p* = 0.003), and a lower prevalence of family history of psychiatric disorders (*p* = 0.015). Importantly, patients in SI group exhibited significantly higher HAMD total scores and PSQI scores (all *p* < 0.001).

**TABLE 1:** Demographic and clinical characteristics of depressed patients with and without suicidal ideation.

	SI (*N* = 49)	N‐SI (*N* = 54)	*t/z/χ2*	*p* value
Age	34.45 ± 12.80	36.28 ± 11.85	0.75	0.453
Sex (female)	23 (46.9%)	30 (55.6%)	0.76	0.382
Duration of current episode (months)	2.58 ± 1.06	2.01 ± 0.86	−3.33	**0.001**
Number of episodes	2.55 ± 1.19	2.07 ± 1.03	−1.98	**0.048**
Past SI history (yes)	35 (71.4%)	23 (42.6%)	8.68	**0.003**
Family history (yes)	5 (10.2%)	16 (29.6%)	5.97	**0.015**
Education (years)			3.14	0.391
Primary school	3	2		
Junior high school	17	21		
High school	19	26		
College or above	10	5		
HAMD scores	30.96 ± 3.21	24.00 ± 3.05	−7.98	**<0.001**
PSQI scores	15.94 ± 2.05	12.50 ± 1.76	−6.42	**<0.001**

**Abbreviations**: HAMD: Hamilton Depression Rating Scale; N‐SI: non‐suicidal ideation; PSQI: Pittsburgh Sleep Quality Index; SI: suicidal ideation.

Values are bolded when *p* value < 0.05.

### 3.2 Sleep Architecture Differences Between SI and N‐SI Patients

Analysis of PSG‐derived sleep architecture and physiological parameters revealed no significant differences between the SI and N‐SI groups in TIB, TST, REM latency, SE, N1%, REM%, NWAK, or AI (all *p* > 0.05). The SI group showed a non‐significant trend toward shorter SL (*p* = 0.060) and higher N2% (*p* = 0.071). Notably, the SI group exhibited a significantly lower N3% (*p* = 0.037) and a higher AHI (*p* = 0.013) compared to the N‐SI group (Table 2).

**TABLE 2:** Comparison of sleep architecture and physiology between the SI and N‐SI groups.

	SI (*N* = 49)	N‐SI (*N* = 54)	*F*	*p* value	Partial *η^2^*
TIB (min)	564.30 ± 81.33	559.49 ± 77.10	0.09	0.770	0.001
TST (min)	489.90 ± 90.08	487.00 ± 106.82	0.11	0.743	0.001
SL (min)	17.38 ± 19.34	24.54 ± 33.98	3.62	0.060	0.037
REM Latency (min)	192.61 ± 110.72	183.13 ± 113.13	0.02	0.887	0.000
SE	86.64 ± 9.75	86.64 ± 12.77	0.26	0.611	0.003
N1%	9.29 ± 5.56	12.08 ± 7.50	0.19	0.662	0.002
N2%	54.20 ± 13.06	48.18 ± 17.79	3.34	0.071	0.034
N3%	23.89 ± 13.45	27.00 ± 15.95	4.47	0.037	0.045
REM%	12.63 ± 7.41	12.97 ± 6.83	1.48	0.226	0.015
NWAK	83.96 ± 56.72	102.96 ± 68.88	0.99	0.322	0.010
AI	10.06 ± 5.60	13.20 ± 9.04	1.78	0.186	0.018
AHI	5.64 ± 6.79	2.25 ± 1.67	6.46	0.013	0.064

**Abbreviations**: AHI: apnea–hypopnea index; AI: Arousal Index; N2: NREM stage 2; N3: NREM stage 3; NI: NREM (non‐rapid eye movement) stage 1; NWAK: total number of awakenings; REM: rapid eye movement; SE: sleep efficiency; SL: sleep latency; TIB: time in bed; TST: total sleep time.

### 3.3 Altered Sleep Characteristics Associated With Suicidal Ideation in MDD

To identify sleep characteristics associated with SI in MDD, a binary logistic regression was performed, adjusting for age, sex, duration of the current episode, number of depressive episodes, history of past SI, and family psychiatric history. Bootstrap‐adjusted regression analysis indicated that higher PSQI scores (OR = 3.10, 95% CI: 0.931 to 1.643, *p* = 0.001) and elevated AHI (OR = 20.94, 95% CI: 1.119 to 7.434, *p* = 0.005) were independent predictors of SI. In contrast, a greater proportion of N3 sleep served as a protective factor (OR = 0.015, 95% CI: –10.91 to –0.280, *p* = 0.045). SL was not significantly associated with SI (*p* = 0.821) (Table 3).

**TABLE 3:** Sleep disturbances associated with suicidal ideation in MDD.

	*β*	BootSE	OR	95% CI		*p* value
PSQI	1.13	0.189	3.100	0.931	1.643	0.001
SL	−0.16	0.926	0.853	−1.880	1.896	0.821
N3 percentage	−4.23	2.787	0.015	−10.91	−0.280	0.045
AHI	3.04	1.550	20.935	1.119	7.434	0.005

**Abbreviations**: AHI: apnea–hypopnea index; N3: NREM stage 3; PSQI: Pittsburgh Sleep Quality Index; SL: sleep latency.

**FIGURE 1:**
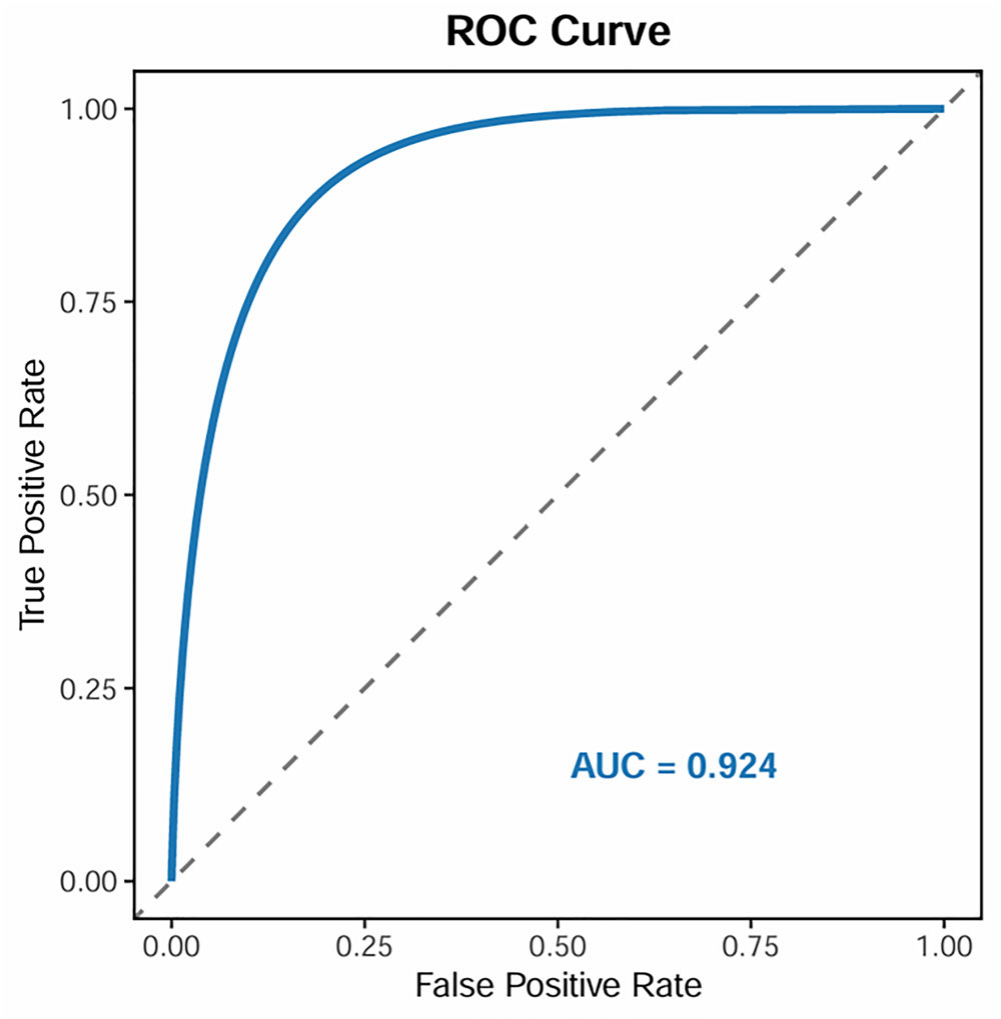
ROC curve for predicting suicidal ideation using PSQI, N3%, and AHI in MDD.

After removing SL from the model, the combined predictive value of PSQI, N3%, and AHI remained strong, with an area under the ROC curve (AUC) of 0.924, indicating excellent discrimination between patients with and without SI (Figure 1).

## 4 Discussion

In this study, we systematically compared sleep architecture and physiological parameters between MDD inpatients with and without suicidal ideation. Despite comparable demographic profiles, patients with SI displayed a significantly more severe clinical presentation, including longer duration of the current depressive episode, higher number of lifetime episodes, and substantially elevated depressive symptoms and subjective sleep disturbances. Critically, objective PSG revealed that specific alterations in sleep architecture, rather than global sleep disruption, were closely associated with SI. Reduced N3 sleep and elevated AHI emerged as robust physiological markers of SI, while subjective sleep disturbance measured by the PSQI remained an independent predictor. Furthermore, logistic regression demonstrated that a combination of PSQI, N3%, and AHI achieved excellent discriminative accuracy between patients with and without SI, underscoring the value of integrating both subjective and objective sleep measures to enhance suicide risk detection in MDD.

Consistent with prior evidence, our results reinforce the strong link between sleep disturbances and SI in MDD (Ballard et al. 2016). Previous studies indicate that subjective sleep complaints, especially poor sleep quality and insomnia symptoms, reliably predict suicidal thoughts and behaviors in both adolescents and adults with MDD (Fan et al. 2024; Gowin et al. 2024). The present findings extend this work by showing that subjective sleep disturbance, as assessed by the PSQI, remains a strong and independent contributor to SI after adjusting for key clinical confounders. While prior PSG studies have reported inconsistent findings regarding which sleep parameters most closely relate to suicidality (Baglioni et al. 2016; Hejazi et al. 2022), our results suggest that differences in sleep architecture, particularly N3 sleep, may be associated with suicidal ideation. The significantly lower N3% observed in the SI group aligns with research linking impaired slow‐wave sleep to emotion dysregulation (Palagini et al. 2013), poor stress recovery (Radwan et al. 2021), and increased vulnerability to suicidal thoughts (Goetz et al. 2001). Regarding REM sleep variables, earlier studies proposed shortened REM latency as a potential biological marker of depression (Kupfer et al. 1986), whereas others emphasized increased REM density (Riemann et al. 1994). In contrast, we found no significant associations between REM sleep parameters and SI. Additionally, the elevated AHI among SI patients supports growing evidence that sleep‐disordered breathing may heighten suicide risk via mechanisms such as intermittent hypoxia, autonomic dysregulation, and increased nocturnal arousal (Bishop et al. 2018; Sweetman and Adams 2022; Udholm et al. 2022). Collectively, these findings emphasize the need for routine and comprehensive sleep assessment in MDD patients and suggest that combined subjective and objective sleep evaluation could improve clinical detection of suicide risk, while sleep‐focused interventions may help reduce SI.

Several neurobiological pathways may explain these associations. First, reduced N3 sleep may reflect diminished slow‐wave activity (Brodt et al. 2023), which is essential for synaptic homeostasis, emotion regulation, and stress recovery (Kamphuis et al. 2015; Liu et al. 2024). Insufficient deep sleep has been associated with heightened amygdala reactivity and diminished prefrontal control (Pereira et al. 2022; Skelin et al. 2021), both of which contribute to affective instability and suicidal ideation (Cong et al. 2022; Zheng et al. 2023). Second, elevated AHI suggests the presence of sleep‐disordered breathing, which may exacerbate neural vulnerability through intermittent hypoxia, oxidative stress, and systemic inflammation (Lim and Veasey 2010; Meliante et al. 2023). Such physiological disturbances are associated with impaired cognitive control and increased impulsivity, which are well‐established risk factors for suicide in MDD (Herzog et al. 2025; Zhang et al. 2025). Third, subjective sleep disturbances captured by the PSQI may reflect perceived sleep‐related distress rather than objective sleep architecture abnormalities (Hartmann et al. 2015), as they are not necessarily explained by polysomnography‐derived sleep measures. Consistent with this, in our dataset, PSQI scores were not significantly correlated with PSG‐derived sleep parameters. This perceived sleep disturbance may foster hopelessness and rumination (Cao et al. 2023), two well‐established psychological mechanisms underlying suicidality (Kovács et al. 2020; Morrison and O'Connor 2008; Pettorruso et al. 2020). Together, these pathways suggest that both objective physiological abnormalities and subjective sleep‐related distress converge to increase suicide vulnerability in MDD.

Several limitations should be noted. Firstly, the cross‐sectional design precludes causal conclusions regarding the directionality of the relationship between sleep disturbances and SI. Longitudinal studies are needed to clarify temporal dynamics. Secondly, although PSG provides objective data, a single‐night recording may not fully represent habitual sleep patterns. In addition, PSG‐derived measures of nocturnal oxygenation (e.g., SpO_2_ nadir and time below 90% oxygen saturation) were unavailable. Given that nocturnal hypoxia has been reported to be associated with anxiety symptoms (Jiang et al. 2025), future studies should incorporate these measures to better characterize intermittent hypoxia. Thirdly, the sample size, while sufficient for primary analyses, may limit generalizability and the detection of smaller effect sizes. Fourthly, assessing SI based on a single HAMD‐17 item may not capture the full complexity of suicidal ideation. Finally, recent evidence has indicated that REM density may be associated with rapid antidepressant response (Kheirkhah et al. 2025); thus, future studies are warranted to investigate its potential clinical relevance.

In conclusion, this study demonstrates that both subjective and objective sleep disruptions, particularly poor sleep quality, reduced deep sleep, and elevated AHI, are strongly associated with SI in MDD. These findings highlight the importance of incorporating sleep assessment into suicide risk evaluation and point to sleep‐related factors as potential targets for therapeutic intervention.

## Author Contributions

**Limin Yang**: conceptualization, data curation, investigation, methodology, Writing – original draft. **Xuefei Wu**: conceptualization, data curation, investigation, methodology, resources, supervision. **Yu Wang**: formal analysis, software, validation, visualization, writing – original draft. **Shilong Jia**: data curation, investigation. **Renyun Zhang**: data curation, investigation. **Yun'ai Su**: formal analysis, software, validation, visualization. **Jitao Li**: conceptualization, funding acquisition, methodology, project administration, supervision, resources.

## Funding

This work was supported by the National Natural Science Foundation of China (grant No. 82471544 and 82271569) and the Capital Medical Development Research Fund (2026‐2‐4113). The funders have no role in study design, data collection and analysis, decision to publish, or preparation of the manuscript.

## Ethics Statement

This study has obtained ethical approval from the Ethics Committee of Shandong Provincial Mental Health Center (Approval No.KYSJWLL2024‐1‐087). All participants provided written informed consent prior to their inclusion in the study. The research was conducted in accordance with the ethical principles outlined in the Declaration of Helsinki.

## Conflicts of Interest

The authors declare no competing interests.

## Data Availability

The datasets used during the current study are not publicly available, but are available from the corresponding author on reasonable request.
